# Linear Growth and Final Height Characteristics in Adolescent Females with Anorexia Nervosa

**DOI:** 10.1371/journal.pone.0045504

**Published:** 2012-09-18

**Authors:** Dalit Modan-Moses, Amit Yaroslavsky, Brigitte Kochavi, Anat Toledano, Sharon Segev, Fadel Balawi, Edith Mitrany, Daniel Stein

**Affiliations:** 1 Pediatric Endocrinology and Diabetes Unit, The Edmond and Lily Safra Children’s Hospital, The Chaim Sheba Medical Center, Tel-Hashomer, Ramat Gan, Israel; 2 Pediatric Psychosomatic Department, The Edmond and Lily Safra Children’s Hospital, The Chaim Sheba Medical Center, Tel-Hashomer, Ramat Gan, Israel; 3 The Sackler School of Medicine, Tel Aviv University, Tel Aviv, Israel; University of South Australia, Australia

## Abstract

**Objective:**

Growth retardation is an established complication of anorexia nervosa (AN). However, findings concerning final height of AN patients are inconsistent. The aim of this study was to assess these phenomena in female adolescent inpatients with AN.

**Methods:**

We retrospectively studied all 211 female adolescent AN patients hospitalized in an inpatient eating disorders department from 1/1/1987 to 31/12/99. Height and weight were assessed at admission and thereafter routinely during hospitalization and follow-up. Final height was measured in 69 patients 2–10 years after discharge. Pre-morbid height data was available in 29 patients.

**Results:**

Patients’ height standard deviation scores (SDS) on admission (−0.285±1.0) and discharge (−0.271±1.02) were significantly (p<0.001) lower than expected in normal adolescents. Patients admitted at age ≤13 years, or less than 1 year after menarche, were more severely growth-impaired than patients admitted at an older age, (p = 0.03). Final height SDS, available for 69 patients, was −0.258±1.04, significantly lower than expected in a normal population (p = 0.04), and was more severely compromised in patients who were admitted less than 1 year from their menarche. In a subgroup of 29 patients with complete growth data (pre-morbid, admission, discharge, and final adult height), the pre-morbid height SDS was not significantly different from the expected (−0.11±1.1), whereas heights at the other time points were significantly (p = 0.001) lower (−0.56±1.2, −0.52±1.2, and −0.6±1.2, respectively).

**Conclusions:**

Our findings suggest that whereas the premorbid height of female adolescent AN patients is normal, linear growth retardation is a prominent feature of their illness. Weight restoration is associated with catch-up growth, but complete catch-up is often not achieved.

## Introduction

Malnutrition or systemic diseases often result in growth deceleration and stunting. While nutritional rehabilitation or disease remittance results in accelerated linear growth, [1]–[3], catch-up growth is often incomplete, resulting in compromised final adult height [1], [4], [5]. Despite the well-described association between nutrition and growth, only few systemized studies assessed the effect of undernutrition during puberty on growth rate and final adult height. Moreover, while catch-up growth is easily recognized in the pre-pubertal period, it is often difficult to discriminate between the accelerated growth rate of the normal pubertal spurt and the occurrence of catch-up growth in nutritionally rehabilitated malnourished pubertal adolescents.

Anorexia nervosa (AN), which affects about 1% of adolescent females, is characterized by an intense fear of gaining weight, self-imposed semi-starvation, weight loss, and amenorrhea [6]. The disease provides an attractive model for studying the effect of caloric restriction during adolescence on growth and final height, since in AN patients undernutrition occurs in the absence of other physical illness, and weight rehabilitation is planned and controlled.

Several studies reported growth failure or short stature in patients with AN [7]–[14] (Table 1) or other forms of voluntary caloric restriction [15]–[17]. Possible contributors to growth retardation in these patients include hypothyroxinemia, hypercortisolemia, hypogonadism [18]–[20], and growth hormone resistance [21]–[23]. However, despite the relatively high prevalence of AN in adolescent females [24]–[25], data regarding final height of AN patients are scarce and inconclusive [2], [10], [13]. Reports describing catch-up growth in AN range from failure to gain any height [7] to complete catch-up growth [2], [7], [12], [15] (Table 1). This apparent discrepancy reflects small patient series, heterogeneous patient populations, variable duration of follow-up, and absence of pre-morbid growth data.

**Table 1:** Previous studies assessing growth and final height of AN patients.

ref	n	Height			
		Premorbid	Dg/admission	Follow-up	Final
2	F = 58, M = 13	na			“As expected”
7	F = 1, M = 2	nl	decreased	1 = catch-up, 2 = na	na
8	F = 13, M = 2	na	decreased	11 pats had catch-up	
9	M = 10	na	decreased	Na	na
10	F = 16	na	decreased	Ht-SDS improved	Near FH <TH
11	M = 11	nl	decreased	Ht-SDS improved	FH<premorbid height SDS and TH- SDS
12	F = 46	nl	decreased	Ht-SDS normalized	na
13	F = 681*, Ctl = 832	na	na	Na	Decreased**
14	F = 63, Ctl79	na	nl	nl growth (1 year)	na
31	F = 58, M = 8	F = nl, M = tall	F = decreased, M = na	F = decreased, M = na	na

Abberiviations: F = female; M = male; Ctl = control; na = not available; pats = patients; FH = final height; TH = midparental target height.

* The mean age of onset in participants with AN was 17.9±3.4 years.

** onset before the age of 16, the analysis showed there was a significant effect of both age of onset and lowest BMI.

We have previously shown that linear growth retardation was common in male adolescent AN patients [11]. Weight restoration was associated with significant catch-up growth, but complete catch up growth was achieved only in a minority of the patients; Although the mean adult final height standard deviation score (SDS) was higher than the admission height SDS, it was lower than both the pre-morbid and the mid-parental target height SDSs [11].

The aims of the present study were to evaluate the prevalence of growth retardation in a large cohort of female adolescent AN inpatients, to assess the effect of weight restoration on catch-up growth and final height, and to identify factors affecting catch-up growth and final height. We hypothesized that adolescent female AN patients would show growth retardation upon hospitalization, and that weight restoration would be associated with significant, although not complete, catch up growth. Since longer duration of AN is associated with greater arrest of growth [14], we expected that patients admitted at a younger age, likely presenting at earlier stages of puberty and with shorter illness duration, will have a greater potential for catch-up growth than patients hospitalized at later pubertal stages.

## Patients and Methods

### Patients

All female AN patients (n = 211), hospitalized in the Pediatric Psychosomatic Department of the Edmond and Lily Safra Children's Hospital from 1/1/1987 to 31/12/1999 were included in this study, and their medical charts were retrospectively reviewed.

The study was approved by the hospital's review board. AN diagnosis as summarized in the medical records was re-assessed independently by two experienced psychiatrists (D.S., A.Y.) according to the criteria of the DSM-IV, using the Eating Disorders Family History Interview (EDFHI) [26] that has been validated in Israeli samples [27].

The patients did not receive any psychotropic medications prior to admission or during the first month of inpatient treatment. Thereafter, antidepressant agents were administered during hospitalization in about 50% of the patients upon achieving a body mass index (BMI) of at least 17 kg/m^2^. While these medications may increase appetite, cause weight gain, and alter endocrine function, to the best of our knowledge, they do not significantly influence linear growth [28].

### Management

After assessment of the nutritional status, a nutritional rehabilitation program geared toward weight-gain of 0.5–1.0 kg/week was constructed. Target weight was established according to age and the estimated potential height. Patients were discharged upon reaching their target weight and maintaining it for at least two weeks.

After discharge, patients were followed biweekly during the first two months, monthly for the next four months, and thereafter, every three months until reaching the age of 18. Target weight was readjusted every three months during follow-up in patients who had not finished growing, and was increased gradually to allow for the expected height gain, based on the potential height.

### Height and Weight Measurements

Standing height was measured to the nearest 0.1 cm, using a wall mounted stadiometer. All measurements were taken during the morning hours by a single investigator (A.T.), using standardized procedures [29]. Body weight was obtained to the nearest 0.1 kg, with the patient wearing a hospital gown and without any footwear.

Pre-morbid height and weight measurements, when available, were obtained from the primary care physician and/or the school nurse.

Final height (defined conservatively as height at age 18 or older, and at least 3 years after menarche) was measured in our center in 69 patients 2–10 years following discharge from their index hospitalization. Height SDSs were calculated based on the 2000 sex-specific growth charts from the CDC (www.cdc.gov/growthcharts). These data have been found adequate for assessing Israeli children [30].

### Data Analysis

Height SDSs at the various time points were compared to the expected in the normal population using the one-sample t-test. Height and weight on admission and discharge were compared using the paired t-test. ANOVA with repeated measures was used for comparing changes in height SDSs over time in patients who had multiple measurements, and in subgroups of patients (younger vs. older). Results were considered significant if the two-sided p-value was <0.05. Calculations were performed using SPSS 14.0, a statistical software package.

## Results

### Baseline Characteristics of the Study Patient (n = 211)

The mean age of the patients on admission was 16.6±4.2, years; their mean BMI was 15.7±1.02 kg/m2, and their mean age at menarche was 12.7±2.4 years.

### Weight Gain during Hospitalization (n = 211)

Patients' weight increased significantly (p<0.001) during hospitalization (5.3±8.2 months). Average weight gain was 7.98±6.43 kg.

### Linear Growth during Hospitalization and Weight Rehabilitation (n = 211)

Mean height SDS on admission was −0.285±1.02, significantly lower than expected in a normal population (p<0.001). During hospitalization, height SDS increased to −0.271±1.02, but was still significantly lower than expected in a normal population (p<0.001).

**Figure 1:**
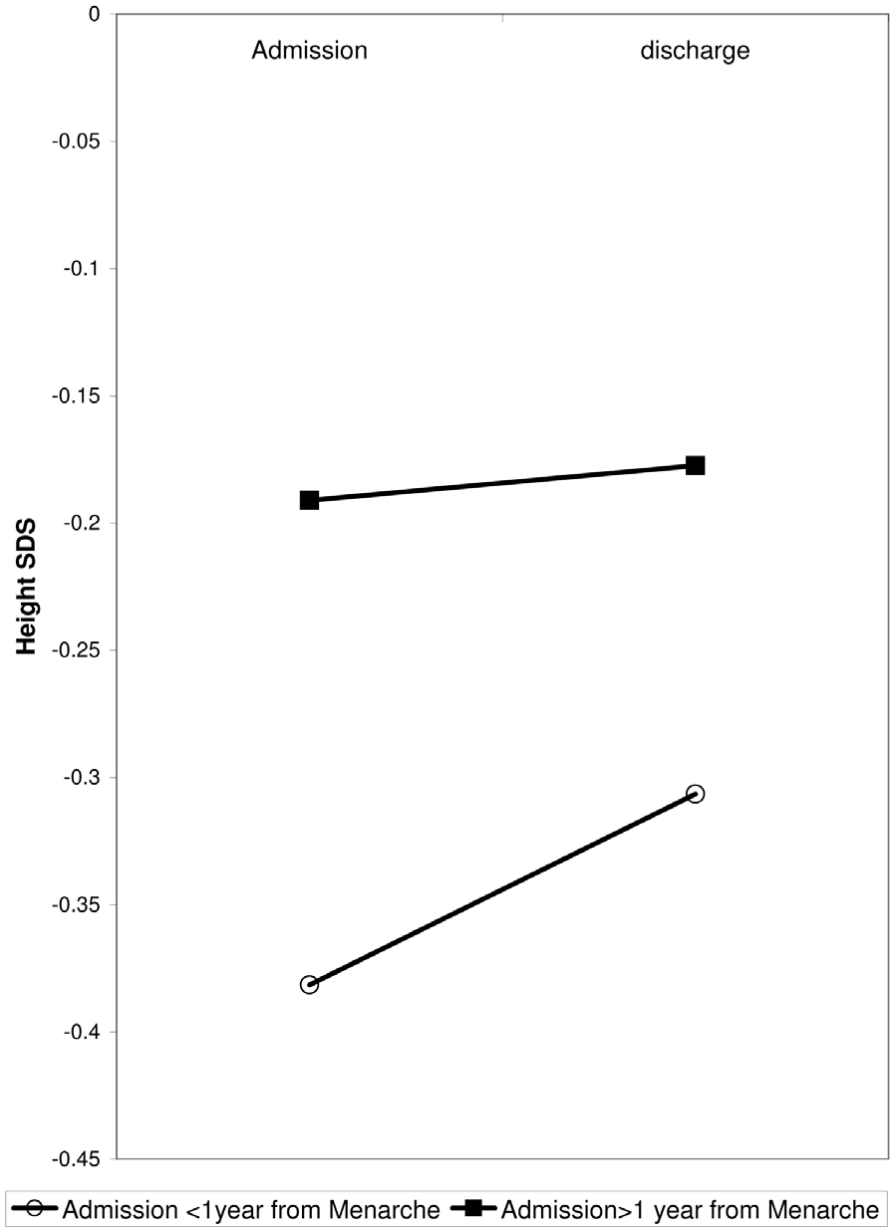
The correlation of pubertal status at admission with height SDS on admission and discharge from hospitalization.

ANOVA with repeated measures revealed that the extent of growth impairment was more severe among patients admitted at an age of 13 years or younger, compared to patients admitted at an older age, on both admission and discharge (p = 0.03). Similarly, ANOVA with repeated measures revealed that patients admitted less than 1 year after menarche had more severe growth-impairment on admission than patients admitted more than 1 year after menarche (height SDS −0.38±1.01 vs. −0.19±0.92, p = 0.03) (figure 1). There was a trend toward greater improvement in height SDS during hospitalization among patients admitted less than 1 year after menarche (figure 1), but this did not reach significance.

### Final Height Data (n = 69)

Final height was available for 69 patients (32.7%). Three of the original cohort of 211 candidates had died (2%; data obtained from the death records of the Israel Ministry of Interior Affairs). Comparison of patients who were evaluated for final height to those who were not, showed no differences for year of birth, ethnic origin, age at the onset of AN and at hospitalization, time from the onset of AN to hospitalization in our department, duration of hospitalization, minimal body mass index (BMI), subtype of AN at admission, and weight gain during hospitalization (data not presented).

On admission, the mean height SDS of these 69 patients was −0.231±1.103, significantly lower than expected in a normal population (p<0.001). During hospitalization, height SDS increased to –0.197±1.122, but was still significantly lower than expected in a normal population (p<0.001). Mean final height SDS was −0.258±1.04 (161.6±6.8 cm), which, similar to the admission and discharge height SDS, was significantly lower than expected in a normal population (p = 0.04).

### Factors Affecting Catch-up Growth and Final Height

Height SDS on admission was a strong predictor of final height SDS (p<0.001). BMI-SDS upon admission was negatively correlated with the change in height-SDS between admission and final height (r = −0.306, p = 0.011). Other factors, including weight gain during hospitalization, duration of hospitalization, number of hospitalizations, and duration of AN prior to hospitalization had no significant affect on final height SDS.

**Figure 2:**
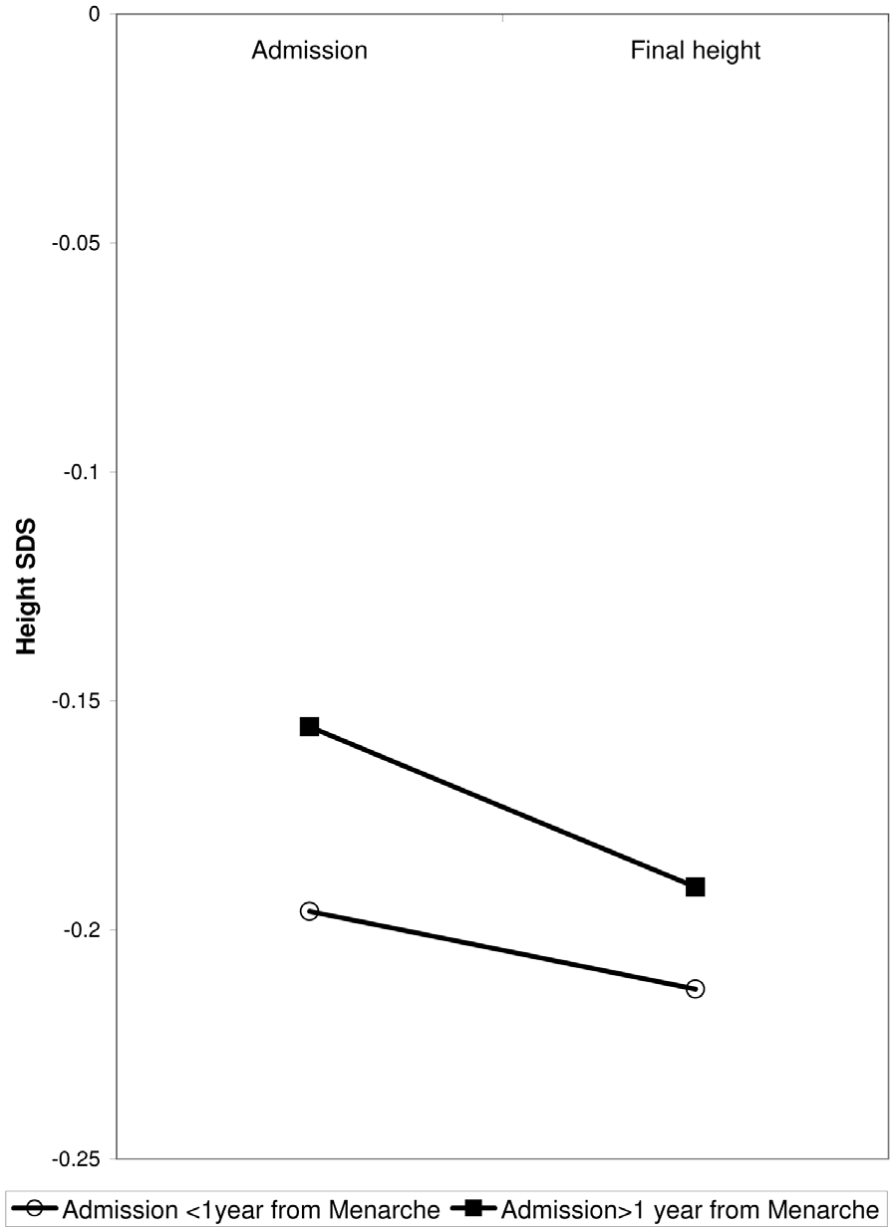
The correlation of pubertal status on admission with final adult height.

To test our hypothesis that patients admitted at earlier stages of puberty will have a greater potential for catch-up growth compared to patients hospitalized at later pubertal stages, we divided the 69 patients for whom final height was available into those who were admitted less than 1 year after menarche and patients admitted more than 1 year from menarche. A significant (p = 0.019) interaction was found between final height and pubertal status at admission. Hence, in contrast to our hypothesis, patients who were admitted less than 1 year after menarche showed less catch-up growth and their final height was more severely compromised in comparison to patients admitted more than one year after menarche (figure 2).

### Comparison of Admission, Discharge, and Final Height with Pre-morbid Growth Characteristics (n = 29)

**Figure 3:**
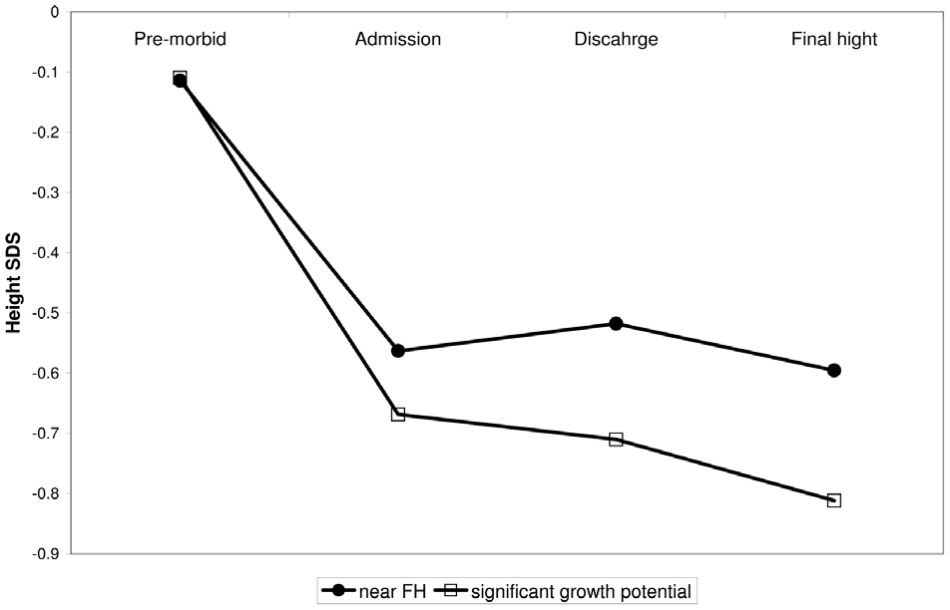
Changes in height SDS in 29 anorexia nervosa patients with complete growth data. This figure presents premorbid height-SDS in comparison with height-SDS at the time of admission and discharge, as well as with final height. The mean pre-morbid height SDS at the time of admission was significantly lower than the pre-morbid height SDS. The final height SDS was lower than both the premorbid and the target height SDS, and higher than the height SDS on admission. (• - Patients judged to be at near final height on admission; □ - Patients judged to have significant growth potential on admission).

Complete growth data (i.e. pre-morbid, admission, discharge, and final height) was available for 29 patients. The mean pre-morbid height SDS of this sub-group was −0.11±1.1, similar to that expected in a normal population. At the time of admission, the mean height SDS was lower, −0.56±1.2, indicating a decrease in linear growth prior to admission. During hospitalization, height SDS increased marginally to −0.52±1.2 but remained lower than the pre-morbid height SDS. The mean final height SDS was −0.6±1.2 (159.3±7 cm), again lower than the pre-morbid height SDS (Figure 3).

ANOVA with repeated measures revealed a significant (p = 0.001) time effect. Using contrasts to compare the various time points revealed that the pre-morbid height-SDS was significantly different from the other three time points, which were all similar. It could be argued that some of our patients achieved final height prior to admission, and therefore had little potential for catch-up growth. We therefore repeated the analysis excluding 14 patients who were likely at or near final height at the time of admission (age>16 years, more than 2 years after menarche).

The results for the remaining 15 patients (age<16 years and less then 2 years after menarche) were similar: premorbid height SDS in this SUB-group (−0.11±1.010) was significantly higher compared to height SDS at admission (−0.67±1.33), at discharge (−0.71±1.3), and at final height −0.81±1.21 (Figure 3).

## Discussion

The aim of the present study was to assess linear growth retardation characteristics in adolescent females with AN, with particular attention to the period post weight restoration. Based on our findings in male patients [11] we hypothesized that female AN patients would show significant growth retardation upon hospitalization, and that weight restoration would be associated with a significant, although not complete, catch up growth. Our results support this hypothesis, as growth impairment was indeed prevalent in our group of adolescent female AN inpatients, from admission until attainment of final adult height.

The mean height SDS on admission for all 211 patients was −0.285±1.02, significantly lower than expected in a normal population. In the subgroup of patients with complete growth data, the mean pre-morbid height SDS was not significantly different from the expected in a normal population, whereas mean height SDS on admission was significantly lower. These results are in agreement with Swenne et. al. and Nielsen [12], [31] (Table 1), and suggest that the decreased height SDS observed on admission reflects actual growth retardation, rather than an increased predilection to AN in females with short stature as suggested elsewhere [13]. Furthermore, our findings imply that early signs of AN may not be detected in a significant number of patients, and that the diagnosis of the disorder is often delayed.

Patients admitted at age 13 years or younger, and patients admitted less than one year after menarche presented with more severe growth-impairment compared to patients admitted at an older age. A possible explanation of this finding is that some of the older patients may have already accomplished most of their linear growth prior to the onset of AN.

Weight restoration resulted in accelerated linear growth and improvement in height SDS during hospitalization. Still, complete catch-up growth was not achieved, and the mean final height SDS of our patients was significantly lower than expected in a normal population, and not significantly different from height SDS on admission. Considering the pre-morbid records, our data suggest that compromised final adult height was the result of prolonged malnutrition and not related to pre-morbid short stature. This notion is further supported by the finding that BMI-SDS upon admission was negatively correlated with the difference between height-SDS on admission and at final height, implying that patients with a greater degree of malnutrition upon admission had a lesser degree of catch-up growth.

Several hormonal changes may contribute to growth retardation in AN. These include low thyroxine and triiodothyronine, elevated cortisol, and low sex hormone levels. Growth hormone (GH) resistance - characterized by GH hypersecretion, and low serum levels of growth hormone binding protein, insulin-like growth factor I (IGF-I), and insulin-like growth factor binding-protein 3 (IGF-BP3) - has also been related to growth failure in AN patients. These changes are reversible with weight gain, with a gradual increase in thyroid hormone levels, gonadotropins and IGF-I, and a decrease in GH and cortisol levels (19, 32). In addition, it has been suggested that leptin may act as a skeletal growth factor and induce longitudinal growth (33). Since leptin levels are low in underweight AN patients and increase with re-feeding (32), it is possible that hypoleptinemia may contribute to growth retardation in AN, whereas the increase in leptin levels with weight restoration may contribute to catch-up.

Several smaller studies [7]–[9], [11], [12] (Table 1) described the occurrence of growth retardation in AN. However, whether growth retardation seen at the time of diagnosis of AN affects final adult height has not yet been established. Two studies suggested that adolescents with AN can realize their height potential. Pfeiffer et al [2] assessed final adult height in 13 males and 58 female AN patients, claiming that most patients reached their expected heights. However, final height percentile was compared with height percentile at diagnosis, with no reference to pre-morbid height. Thus, height deceleration prior to admission could have been missed. Moreover, since only limited information was given regarding the patients' age distribution, it is possible that at least some of them reached final height prior to the onset of AN. Prabbhakaran et al [14] followed 63 female AN adolescents for one year. They found that height at study entry, as well as the difference between target height and both follow-up height and predicted adult height did not differ between patients and controls, suggesting the preservation of height potential in female AN adolescents. However, no information was given regarding the premorbid height of the AN patients, and final height was not assessed. Furthermore, this study included patients treated as outpatients that might have had a milder disease course compared to the inpatients included in our study. In keeping with this assumption, the investigators reported that girls with longer duration of illness had lower height SDSs and lower predicted height SDSs [14].

By contrast, the results of Lantzouni et al [10] in 16 females with early onset AN, and of Lanes and Soros [17] in healthy children after prolonged period of inadequate caloric intake, were similar to ours. Both groups reported decreased height SDS at presentation. Despite accelerated growth following nutritional rehabilitation, the patients did not achieve their predicted final height [17] or their target height [10], [17].

**Figure 4:**
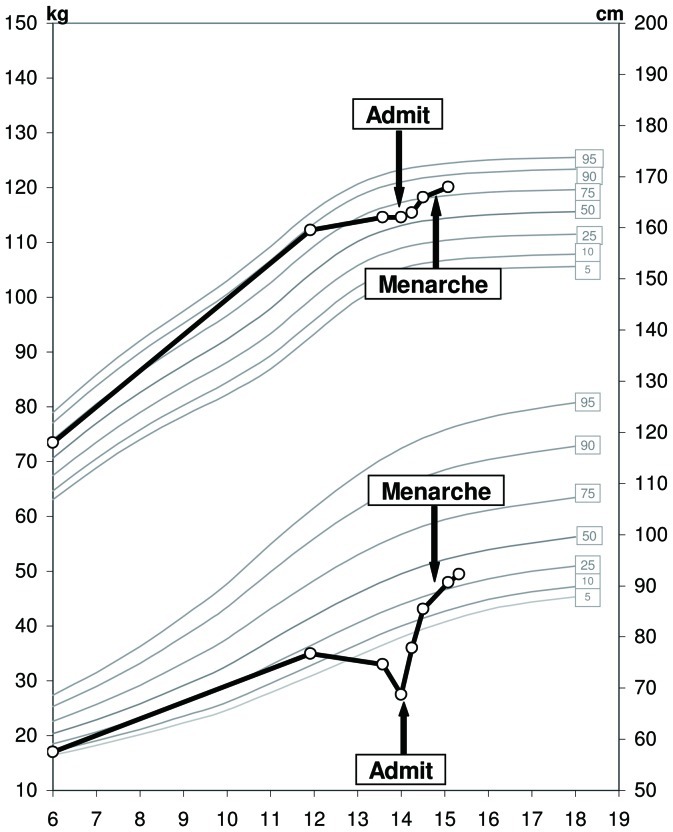
Growth curve of a representative patient. Growth failure (“shifting” of height percentiles) was evident for about 2 years prior to admission. The weight of this patient was at the 25th percentile at age 12 years, and had shifted to considerably below the 3rd percentile during the two years prior to hospitalization. She had virtually no linear growth during the 2 years preceding her admission, with her height decreasing from the 90th percentile to the 50th percentile. Thus, her apparent “normal” height at the time of admission, may reflect growth failure related to delayed diagnosis. Growth deceleration may not have been noted, because height was at or above the 50th percentile, and the patient was probably still perceived as normal compared to her peers. Weight rehabilitation resulted in catch-up growth.

As emphasized earlier, the occurrence of growth impairment in our patients well before hospitalization suggests a considerable delay in the diagnosis of AN. We hypothesize that weight loss in our patients may have been masked by their reduced height: once growth retardation occurs, the severity of malnutrition may be underestimated because weight is related to the “stunted” height rather than to the pre-morbid projected height, resulting in a weight-per-height/BMI SDS that may seem reasonable. In addition, growth impairment may have not been recognized in adolescents whose heights were in the upper percentiles. This is illustrated in figure 4 - the patient depicted in this figure shifted from the 90^th^ to the 50^th^ height percentile well before AN has been noticed, perhaps because she still appeared normal relative to her peers (Fig. 4). Irrespective of the reason, a delay in the diagnosis of AN in adolescent, and perhaps even more so in pre-adolescent females, may result in severe malnutrition and compromised final height. Furthermore, since AN may be associated with irreversible multi-organ damage including brain atrophy [34], [35], osteoporosis [36], and major adverse obstetric outcomes [37], we postulate that stunting of growth may be viewed as a measurable marker of covert tissue injury. Thus, we believe that early diagnosis of AN and early aggressive nutritional rehabilitation are of utmost importance in order to prevent not only growth failure but also irreversible tissue damage with long-term implications.

It has been suggested that the hypogonadism found in AN may lead to delay in growth-plate closure [14], allowing, in turn, for compensatory growth with the potential for catch-up during later adolescence. Hence, we hypothesized that patients admitted at a younger age, with seemingly significant growth potential, will show the greatest degree of catch-up growth. However, in contrast to our hypothesis, and similar to the findings of Favaro et al [13], these patients actually had the worst outcome regarding final height. Thus, it seems that prolonged undernutrition, arising (albeit not diagnosed) during early adolescence and continuing around the critical time of peak height velocity, may interfere with growth and result in irreversible stunting despite the delay in skeletal maturity.

Our series is one of the largest series describing growth in female AN patients. It provides important pre-morbid, follow-up and final-height data not previously available in other series. Nevertheless, several limitations should be considered. Our study included only inpatient AN female adolescents, so that our findings cannot be generalized to less severe forms of the disorder. Secondly, we did not include matched controls. Thirdly, only a third of the original cohort had final height data, and premorbid height was reported in only a minority, although no difference was shown between patients with and without final height data in any of the demographic and clinical parameters evaluated. Although we made an attempt to contact all former patients, many refused to come for a final assessment, or have not been located. This was likely the result of our department being a tertiary care center that hospitalizes patients from all over Israel, reducing the likelihood of continuation of treatment in our center following discharge. Finally, Tanner stages of sexual development, bone age studies, parental heights, and results of laboratory tests were not recorded in most of the charts. Therefore, we were unable to calculate the pre-morbid predicted adult height and the mid-parental target height, or to investigate the relationships between pubertal stages and adult height.

Despite these limitations, our study provides strong evidence to support our hypothesis. In particular, the strength of our study relies on the data obtained from the subgroup of patients with pre-morbid and final height measurements (figure 3), showing normal pre-morbid height followed by growth deceleration and almost no catch-up growth. These patients present an unusual growth pattern that cannot be explained based on genetic height potential or timing of puberty.

It could be argued that growth deceleration and the subsequent catch-up growth observed in our patients may actually represent the effects of delayed puberty per-se. However, inspection of the growth data makes this unlikely. First, the severity of growth failure observed in some of our patients far exceeds the pre-pubertal growth deceleration observed in children with delayed puberty, which is in the range of 4.0–4.5 cm/year [38], [39]. For instance, the patient depicted in figure 4 had virtually no linear growth for the 2 years preceding her hospitalization (figure 4). Secondly, whereas patients with delayed puberty may not reach their parental target height [40], [41], they typically regain or even exceed their own childhood growth percentile [42]. In contrast, in our patients, catch-up growth was incomplete, and their pre-morbid height percentile was not reached.

In conclusion, our findings emphasize the importance of early detection of AN in female adolescents, as growth retardation occurring during a critical growth period during puberty may be irreversible. Hence, AN should be suspected in every adolescent female showing decelerated linear growth. Secondly, in keeping with previous studies [3], [11], [42], we suggest that weight restoration geared towards restitution of height to the pre-morbid percentile for age should be initiated as early as possible in the treatment of AN adolescents. We hypothesize that in order to achieve maximal catch-up growth, pre-morbid growth data should be obtained, and target weight should be based on the expected, rather than the measured height percentile at the time of diagnosis. Acceptance of weight as appropriate for height as measured at admission, with no consideration of the pre-morbid or potential height, may result in perpetuation of growth retardation, leading eventually to short stature. Larger scale prospective linear studies, including sexual maturation evaluation and endocrine function tests, are required to verify our observations, to further define factors influencing final height, and to establish the optimal therapeutic approach for optimizing catch-up growth.
